# Free Use of Tooth-Brushes Essential for Healthful Preservation of Teeth and Gums

**Published:** 1901-04

**Authors:** B. F. Arrington

**Affiliations:** Goldsboro, N. C.


### FREE USE OF TOOTH-BRUSHES ESSENTIAL FOR HEALTHFUL PRESERVATION OF TEETH AND GUMS.

BY DR. B. F. ARRINGTON, GOLDSBORO, N. C.

Now and then we see healthy mouths (teeth and gums), but they are rare exceptions, not more than two or three cases in a thousand, possibly, in which there seems to be no need for use of tooth-brush or other means to keep the teeth and gums in a normal state. Such exceptions are not confined to any nationality, race, type, or grade in society. We may safely venture to assert as a fact that mouths (teeth and gums), as a whole, are almost universally in an abnormal state from childhood to old age, and, coupled with this, it is a lamentable fact that since the establishment of the first dental college in this country to the present time, when there are forty or more, and dentists, twenty-six thousand at least, located and practising in every section of our country from Maine to Mexico, there has been no cessation of the diseases of the teeth and gums, nor effort made to any great extent for check of causes.

To treat and patch up the ravages of disease as it presents, remove the natural and substitute artificial has been the general line of practice, and so continues without much effort to make preventive treatment effective. Much time, energy, and manipulative skill has been appropriated to repair treatment, with inexcusable neglect of *preventive* treatment, and it is to-day the general order of practice in all sections and with the most enlightened and prominent in the profession, and with those who so strenuously contend for, and can boast of, "higher education," as with those less fortunate in educational advantages.

To reverse this order of things, and first advise and administer preventive treatment and follow when necessary with repair and substitute treatment as an alternative, would be a wiser policy and doubtless would prove a more effective means of preserving the natural teeth and keeping the soft tissues and alveolar process in a normal-like state. It is an old saying, and true as gospel, and appropriately applicable to the practice of dentistry, that "an ounce of prevention is worth a pound of cure."

If Dr. W. D. Miller's germ theory pertaining to caries of teeth, which I believe is pretty generally accepted, is true, it is good cause why general interest should be aroused with liberal advocacy for the universal daily free use of tooth-brushes, for it is evident, taking a practical, common-sense view of the subject, that with the free use of brush and water a more perfect dislodgement and removal from the mouth of all germ products of every type can be successfully accomplished than with any or all the germicidal remedies that have been so lavishly rushed upon the market since the promulgation of the germ theory was first published.

The use of tooth-brushes for preservation of teeth and to keep the associate soft tissues in a comparatively normal state is indispensable, and when the right make and quality of brushes are rightly used the disease-checking and preservative results are perceptible and satisfactory beyond questioning or controversy. If the cause of disease is not obliterated, disease certainly is held in check and the necessity for extravagant dental operations are greatly diminished. To get back to first principles and aid nature on simple lines of preventive treatment for preservation of teeth is our first duty; then to bring in play our best skill and highest professional attainments (manipulative) when necessary to repair and substitute. Such procedure strictly followed for a generation or two, or less time possibly, will bring results that may greatly diminish the need of dentists, but the supply will be equal to the demand. The labor in practice will be lightened, expense in service and for service curtailed, more teeth preserved, and dentistry as a profession of a specialty will be more exalted and useful.

The indiscriminate selection and use of tooth-brushes as we find them on the market is more *hurtful* than *beneficial* to teeth or gums. On an average, not one brush in fifty found on the market, principally in drug-stores, is practical in structure and quality of bristle for effective application for thorough cleansing of teeth without detriment to the soft tissues, therefore not desirable nor admissible for daily use. The fault lies somewhere, and the indifference and neglect in the production of tooth-brushes will continue, and possibly increase, if investigation is not made and improvement encouraged and demanded. More consideration and careful study should be given to the subject. The work should commence and be continued in dental colleges, and dentists generally should study and discuss the subject freely for mutual benefit, and talk freely in an educational way with patrons and demonstrate to them the necessity for the free use of tooth-brushes, pointing out plainly wherein benefit is to be derived by the use of brushes, commencing with early childhood. Practical education in the use of the brush is requisite, and must be imparted before full benefit can be derived. The results attainable will amply compensate for the expenditure of time and effort requisite for proper instruction.

Holding to the idea, as I do, that there is much reckless extracting of teeth and an unnecessarily large number of teeth being filled and crowned, and that there are possible means for prevention of such practice to a large extent, with increased benefit to teeth, I feel it to be my duty and the duty of all dentists to make every effort possible for such a result. For success on this line, improvement in pattern and production of brushes is essential, and the increased right use of them must follow, commencing systematically with the period of childhood and continuing as a daily practice, that good may be accomplished for the healthful preservation of teeth and greatly lessen necessity for the use of scalers, pluggers, and forceps.

Large-sized tooth-brushes with bristle tufts closely set are very objectionable and never should be used. There can be no reasonable argument offered to justify the use of such a type of brush, yet there are many of them on the market, and are highly recommended by some dentists, and are freely used, with injury to gums and no benefit to teeth. There are many patterns of brushes and grade of bristles in use that are seriously objectionable, and should be condemned and discarded.

I will ask, Would it not be well for dentists to advise with and instruct manufacturers of tooth-brushes, supply dealers, and druggists on the subject, point out to them the *practical* and *impractical* features of the brushes on the market, and inform them of the needs of dentists and the public, a good line of practical, effective, tooth-preserving tooth-brushes?

It would be a humane service, and if heeded would prove a reliable and saving help in treatment for health and preservation of teeth and gums. There has been and is at present too much indifference and too little thought given to the subject.

More practical tooth-brushes and a more liberal and general use of them, with systematic use of toothpicks made of quill, whalebone, or wood, better shaped than those in general use, would greatly diminish caries in teeth, formation of deposits, and disease of the gums.

The chief object in result in the use of tooth-brushes is to cleanse the teeth and free them of impacted substances and excess of germ products, and not, as many seem to think and practise, simply to whiten and beautify the surfaces of teeth exposed to view. Education must correct the error.

A tooth-brush for effectiveness should not be extreme in any feature, but better be too small than too large. The following outline of pattern of brush is simple and practical, and has given perfect satisfaction when used. From fifteen to twenty tufts of bristles in a brush is ample for effective use. The bristles should be of medium stiffness and from one-half inch to nine-sixteenths inch in length, bristles as set in brush seven-sixteenths of an inch in width, and from one and one-quarter to one and one-half inches in length, with space between rows across the brush from three-eighths to one-quarter of an inch each, brush slightly pointed, with bunch of tufts of bristles near the end trimmed to a point, and each row of bristles serrated one-third or half the length of tufts. A brush so constructed and proportioned might be termed a self-cleansing brush. It is always free of impacted accumulations, and can by proper manipulation be made to reach easily every division of tooth surface, fissures, and indentations possible to be reached and cleansed with a brush.

The use of such a brush in moderation, from three to five times daily, at rising and retiring and after each meal, requiring not more than one minute of time for each brushing, will prove a wholesome and profitable practice, and should be systematically indulged when possible. It is all-important that a reasonable quantity of water shall be held in the mouth while exercising use of brush: it aids materially in dislodging and removing successfully all impacted accumulations and excess of germ products, bacteria, microbes, etc. To follow the use of the brush with finger-pressure on gums is a good practice: it tends to keep the gums firm and healthy.

The brush and water practice with the aid of finger-pressure is preventive treatment, simple and practical, and is a surer means and guarantee of results desired, well-preserved teeth and gums, and freedom from unwholesome taint of breath, than can be effected by any other line of treatment. The idea that the mouth is a cesspool of disease-producing germs, that requires for holding in check the daily and hourly use of some of the many *medicinal remedies* now in use and so extravagantly endorsed, is an absurd feature in theory and practice, and should not be encouraged. It is out of reason and is not sustained by good results perceptible with teeth or gums.

This to some may appear a small subject (tooth-brushes), but it is an important one in the interest of health and comfort, and requires that much thought shall be given to it and much shall be said about it that good may be accomplished, not in the interest of a few, but many, of the human family. All are liable to suffer from an abnormal state of the teeth and gums through neglect.

As evidence of the beneficial effects of the use of tooth-brushes let us inspect carefully the condition of the teeth and gums of persons (old and young) who practise daily the use of the brush, and contrast with the condition of those who neglect and ignore the use of it: the difference in result is plain and convincing, and should stimulate thought and advocacy of effort for a more general use of tooth-brushes,—a practice which, if strictly observed, would in all probability, in a limited period of time, greatly lessen the necessity for extreme dental operations for the preservation of teeth. As preservative of the teeth, so with the gums; the gums would seldom be diseased to the extent of requiring treatment as is now administered. It is seldom the case that we ever find a well-defined typical case of Riggs's disease (pyorrhœa alveolaris) in the mouth of a person who indulges the systematic daily use of the tooth-brush. Another instructive and convincing fact is, that in communities and families educated in the use of the toothbrush and who make free use of them, the above-named disease is of less frequent occurrence than in communities and families where the use of the brush is neglected. I have for some years past given much attention to the treatment of said disease, and can say truthfully that I have no recollection during the past ten years of seeing a bad case of the disease in the mouth of a person of any age, male or female, who practised the use of the tooth-brush several times daily. Such facts are pointers, and merit consideration. Careful study on such lines, with a reasonable amount of experimental work for convincing facts, would possibly enable us to do much for the preservation of teeth and keep the gums in a comparatively normal state.

Until effort is made, intelligently and upon *conservative* lines, we will never know what can be accomplished with tooth-brushes for the preservation of teeth and gums. There is work before us, and the field is broad, that all may work that will.

In our experimental work to preserve teeth without filling and crowning to the extent now practised, and to check pyorrhœa alveolaris in its incipiency and keep the gums in a healthy state, and keep germ products in check without the aid of the daily use of antiseptics and disinfectants, we must keep in mind the fact that such results accomplished will be more truly scientific, more in line of duty and progress, and more meritorious than the present-day practice of inserting large display fillings and crowning and the sundry modes of treating pyorrhœa alveolaris.

Pluggers and scalers are valuable implements, and the use of them essential when needed in treating for preservation of teeth, but to spare them when possible and legitimate is a duty that should be strictly observed.

The distinguished Dr. Miller, who is regarded as the highest authority pertaining to germ products and their influence upon diseases (numerous), stated, as much as a decade back, that "during the last few years the conviction has grown continually stronger, among physicians as well as dentists, that the human mouth, as a gathering-place and incubator of diverse pathogenic germs, performs a significant *rôle* in the production of various disorders of the body, and that if many diseases whose origin is enveloped in mystery could be traced to their source, they would be found to have originated in the oral cavity." Not least among the diseases mentioned as caused by germ products generated in the mouth are caries and pyorrhœa alveolaris, with the evil results of which, when neglected, all of us are familiar, and knowing, as we do, the difficulty and cost of treating successfully when well advanced, we should combine in effort to prevent or check progress in the stages of incipiency, which is possible and can be most successfully accomplished by daily care of the mouth in the free use of tooth-brush and water.

